# Polyhydroxylated Steroids from the Bamboo Coral *Isis hippuris*

**DOI:** 10.3390/md9101829

**Published:** 2011-10-10

**Authors:** Wei-Hua Chen, Shang-Kwei Wang, Chang-Yih Duh

**Affiliations:** 1 Department of Marine Biotechnology and Resources, National Sun Yat-sen University, Kaohsiung 804, Taiwan; E-Mail: x_x1224@yahoo.com.tw; 2 Department of Microbiology, Kaohsiung Medical University, Kaohsiung 807, Taiwan; 3 Centers for Asia-Pacific Ocean Research and Translational Biopharmaceuticals, National Sun Yat-sen University, Kaohsiung 804, Taiwan

**Keywords:** octocoral, *Isis hippuris*, anti-HCMV activity

## Abstract

In previous studies on the secondary metabolites of the Taiwanese octocoral *Isis hippuris*, specimens have always been collected at Green Island. In the course of our studies on bioactive compounds from marine organisms, the acetone-solubles of the Taiwanese octocoral *I. hippuris* collected at Orchid Island have led to the isolation of five new polyoxygenated steroids: hipposterone M–O (**1**–**3**), hipposterol G (**4**) and hippuristeroketal A (**5**). The structures of these compounds were determined on the basis of their spectroscopic and physical data. The anti-HCMV (human cytomegalovirus) activity of **1**–**5** and their cytotoxicity against selected cell lines were evaluated. Compound **2** exhibited inhibitory activity against HCMV, with an EC_50_ value of 6.0 μg/mL.

## 1. Introduction

**Figure 1:**
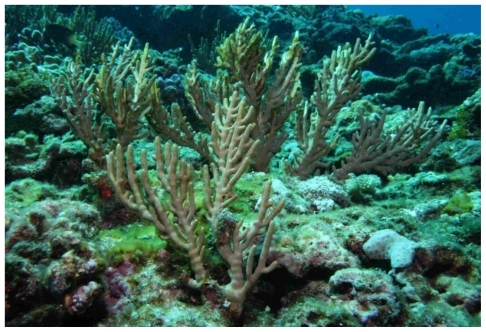
Bamboo coral *Isis hippuris*.

**Figure 2:**
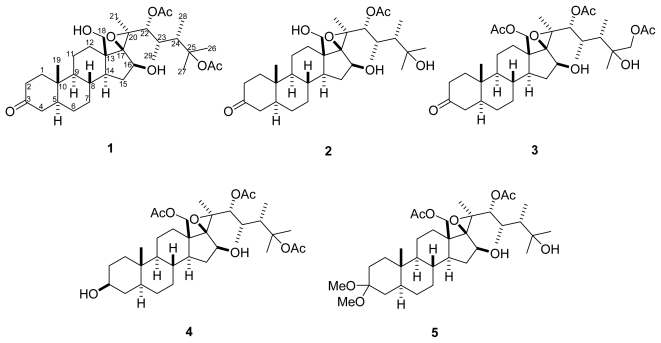
Structures of compounds **1**–**5**.

The octocoral *Isis hippuris*, distributed widely in the western Pacific, has yielded a number of polyoxygenated steroids, including hippuristanol type [1–9], gorgosterol type [10–14], hippuristerone type [3,14,15], and hippuristerol type [3,14–16]. Those of the first type were originally reported as cytotoxins and later rediscovered as selective inhibitors against the translation factor eIF4A [17,18]. Some of the second types were reported to show cytotoxicity or a reversal of multidrug resistance activity [10]. The samples for previous studies on the secondary metabolites of Taiwanese octocoral *I. hippuris* were all collected at Green Island [5–7,12,14,15]. In our continued study of the bioactive metabolites from marine organism, the Taiwanese octocoral *I. hippuris* (Figure 1) collected at Orchid Island was selected for study since its acetone extract exhibited antiviral activity against HCMV. Bioactivity-guided fractionation resulted in the isolation of five polyoxygenated steroids: hipposterone M–O (**1**–**3**), hipposterol G (**4**), hippuristeroketal A (**5**) (Figure 2). We describe herein the isolation, structure elucidation, and biological activity of these compounds.

## 2. Results and Discussion

**Figure 3:**
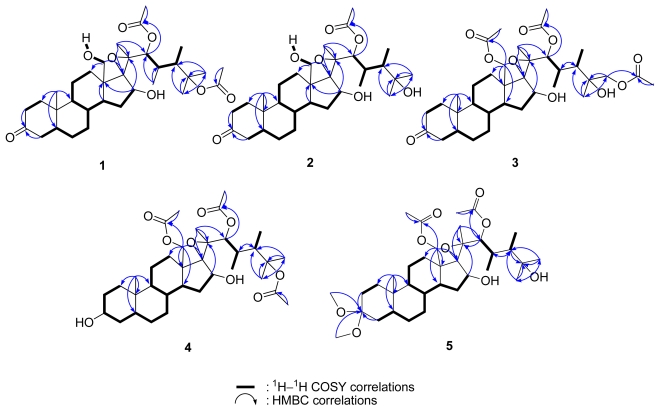
COSY and HMBC correlations of compounds **1**–**5**.

**Figure 4:**
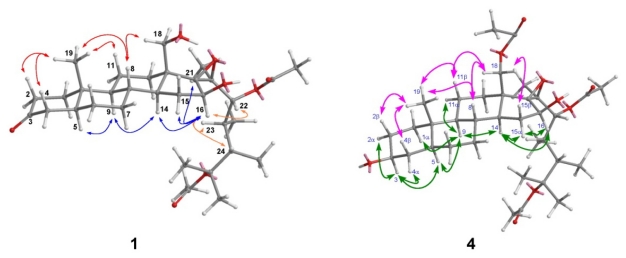
NOESY correlations of compounds **1** and **4**.

The molecular formula C_33_H_52_O_8_ was assigned to hipposterone M (**1**) on the basis of positive HRESIMS (found *m*/*z* 599.3556 [M + Na]^+^), implying eight degrees of unsaturation. Its IR spectrum revealed the absorptions for hydroxyl (ν_max_ 3454 cm^−1^), ketone carbonyl (ν_max_ 1717 cm^−1^), and ester carbonyl (ν_max_ 1733 cm^−1^) groups. NMR data (Tables 1 and 2) of **1** indicated the presence of a ketone (*δ*_C_ 211.7), two ester cabonyls, two oxygenated sp^3^ methines, an oxygenated sp^3^ methylene, three oxygenated sp^3^ quaternary carbons, two secondary methyls, four tertiary methyls, six non-oxygenated sp^3^ methines, eight non-oxygenated sp^3^ methylenes, and two non-oxygenated sp^3^ quaternary carbons. NMR signals (Table 1) at *δ*_C_ 80.0 (qC) and 67.1 (qC) suggested the existence of a tetrasubstituted expoxy. The quaternary carbon at *δ*_C_ 85.5, which has HMBC correlation (Figure 3) with tertiary methyl signals at *δ*_H_ 1.56 (s) and 1.43 (s) (Table 2) disclosed the presence of –OC(CH_3_)_2_. By extensive analysis of 2D NMR spectra, including COSY, HSQC, NOESY (Figure 4) and HMBC, **1** was shown to be a derivative of hippuristerone A [15]. HMBC correlations (Figure 3) from H_2_-18 (*δ*_H_ 3.94 and 3.75) to C-12, C-13, C-14, and C-17 established **1** as 18-hydroxyhippuristerone A. The stereochemistry of the side chain moiety was determined by comparison of the ^1^H and ^13^C NMR spectral data with those of hippuristerone A.

Hipposterone N (**2**) had a molecular formula of C_31_H_50_O_7_, as suggested by the NMR and HRESIMS data. Its IR spectrum also showed the absorptions for hydroxyl (ν_max_ 3454 cm^−1^), ketone carbonyl (ν_max_ 1715 cm^−1^), and ester carbonyl (ν_max_ 1731 cm^−1^) groups. NMR data (Tables 1 and 2) of **2** revealed the presence of a ketone (*δ*_C_ 211.7), an ester cabonyl, two oxygenated sp^3^ methines, an oxygenated sp^3^ methylene, three oxygenated sp^3^ quaternary carbons, two secondary methyls, four tertiary methyls, six non-oxygenated sp^3^ methines, eight non-oxygenated sp^3^ methylenes, and two non-oxygenated sp^3^ quaternary carbons. NMR data (Tables 1 and 2) (Figure 3) of **2** resembled those of **1** except for a hydroxyl group replacing the tertiary acetoxyl in **1** [14]. HMBC correlations (Figure 3) from H_3_-26 (*δ*_H_ 1.24) and H_3_-27 (*δ*_H_ 1.21) to C-25 established **2** as a 25-deacetyl-18-hydroxy derivative of hippuristerone A. The stereochemistry of the side chain moiety was determined by comparison of the ^1^H and ^13^C NMR data with those of hippuristerones F, H, and I isolated from *I. hippuris* [16].

The positive HRESIMS of hipposterone O (**3**) established a molecular formula of C_35_H_54_O_10_. NMR data (Tables 1 and 2) of **3** showed the presence of a ketone (*δ*_C_ 211.5), three ester cabonyls, two oxygenated sp^3^ methines, two oxygenated sp^3^ methylene, three oxygenated sp^3^ quaternary carbons, two secondary methyls, three tertiary methyls, six non-oxygenated sp^3^ methines, eight non-oxygenated sp^3^ methylenes, and two non-oxygenated sp^3^ quaternary carbons. By comparison of NMR spectroscopic data (Tables 1 and 2) of **3** with those of hippuristerone J [14], the primary acetoxy group at C-21 was shift to C-18 on the basis of HMBC correlations (Figure 3) from H_2_-18 [*δ*_H_ 4.23 (1H, d, *J* = 11.6 Hz) and 4.30 (1H, d, *J* = 11.6 Hz)] to C-12, C-13, C-14, C-17, and carbonyl carbon of 18-OAc. The stereochemistry of the side chain moiety was determined by comparison of the ^1^H and ^13^C NMR spectral data with those of hippuristerones J and K previously isolated from *I. hippuris* [14].

Hipposterol G (**4**) was isolated as a white powder, and its molecular formula, C_35_H_56_O_9_, was determined by HRESIMS. Its IR spectrum revealed the functionalities of hydroxyl (ν_max_ 3471 cm^−1^) and ester carbonyl (ν_max_ 1734 cm^−1^). NMR data (Tables 1 and 2) of **4** indicated the presence of three ester cabonyls, three oxygenated sp^3^ methines, an oxygenated sp^3^ methylene, three oxygenated sp^3^ quaternary carbons, two secondary methyls, four tertiary methyls, six non-oxygenated sp^3^ methines, eight non-oxygenated sp^3^ methylenes, and two non-oxygenated sp^3^ quaternary carbons. NMR data (Tables 1 and 2) of **4** were similar to those of hippuristerone G [16] with the absence of the ketone carbon signal at *δ*_C_ 211.6 ppm and the presence of signal at *δ*_H_ 3.60 ppm NOE correlation H-3/H-5 and chemical shift values for C-1–C-7 nuclei. This is in agreement with the results reported for 5α-cholestan-3β-ol, which allowed us to propose a β orientation of OH group at C-3 (Figure 4). The stereochemistry of the side chain moiety was determined by comparison of the ^1^H and ^13^C NMR spectral data with those of hippuristerone A.

The molecular formula of hippuristeroketal A (**5**) was found to be C_35_H_58_O_9_, as deduced from HRESIMS data. Its IR spectrum revealed the absorptions for hydroxyl (ν_max_ 3471 cm^−1^) and ester carbonyl (ν_max_ 1731 cm^−1^) groups. NMR data (Tables 1 and 2) of **5** indicated the presence of a ketal (*δ*_C_ 100.7), two ester cabonyls, two oxygenated sp^3^ methines, an oxygenated sp^3^ methylene, three oxygenated sp^3^ quaternary carbons, two secondary methyls, four tertiary methyls, six non-oxygenated sp^3^ methines, eight non-oxygenated sp^3^ methylenes, and two non-oxygenated sp^3^ quaternary carbons. By comparison of the NMR spectroscopic data (Tables 1 and 2) of **5** resembled those of hippuristerone F [14] with the absence of ketone carbon at *δ*_C_ 211.6 and the presence of two methoxyl signals [*δ*_H_ 3.12 (3H, s), 3.02 (3H, s) and *δ*_C_ 47.6 (CH_3_), 47.5 (CH_3_)] in the molecule. The HMBC correlations (Figure 3) of the methoxyl protons with C-3 [*δ*_C_ 100.7 (qC)], suggesting that C-3 was substituted by two methoxy groups. The stereochemistry of the side chain moiety was determined by comparison of the ^1^H and ^13^C NMR spectral data with those of hippuristerones F, H, and I previously isolated from *I. hippuris* [16]. Compound **5** was not an artifact because ^1^H NMR signals for the dimethylketal were observed before MeOH treatment.

Metabolites **1**–**5** were not cytotoxic against P-388 (mouse lymphocytic leukemia), HT-29 (human colon adenocarcinoma) tumor cells, and human embryonic lung (HEL) cells with IC_50_ values greater than 50 μg/mL. The anti-HCMV activity and cytotoxicity against of selected cell lines of **1**–**5** were evaluated. Compound **2** exhibited inhibitory activity against HCMV, with an EC_50_ values of 6.0 μg/mL.

## 3. Experimental Section

### 3.1. General Experimental Procedures

Optical rotations were determined with a JASCO P1020 digital polarimeter. Ultraviolet (UV) and infrared (IR) spectra were obtained on JASCO V-650 and JASCO FT/IR-4100 spectrophotometers, respectively. NMR spectra were recorded on a Varian MR 400 NMR spectrometer at 400 MHz for ^1^H and 100 MHz for ^13^C or on a Varian Unity INOVA 500 FT-NMR spectrometer at 500 MHz for ^1^H and 125 MHz for ^13^C, respectively. ^1^H NMR chemical shifts are expressed in *δ* (ppm) referring to the solvent peaks *δ*_H_ 7.27 and 7.15 for CDCl_3_ and C_6_D_6_, respectively, and coupling constants are expressed in Hz. ^13^C NMR chemical shifts are expressed in *δ* (ppm) referring to the solvent peaks *δ*_C_ 77.0 and 128.0 for CDCl_3_ and C_6_D_6_, respectively. ESI-MS were recorded by ESI FT-MS on a Bruker APEX II mass spectrometer. Silica gel 60 (Merck, Germany, 230–400 mesh) and LiChroprep RP-18 (Merck, 40–63 μm) were used for column chromatography. Precoated silica gel plates (Merck, Kieselgel 60 F_254_, 0.25 mm) and precoated RP-18 F_254s_ plates (Merck) were used for thin-layer chromatography (TLC) analysis. High-performance liquid chromatography (HPLC) was carried out using a Hitachi L-7100 pump equipped with a Hitachi L-7400 UV detector at 220 nm together with a semi-preparative reversed-phase column (Merck, Hibar LiChrospher RP-18e, 5 μm, 250 × 25 mm).

### 3.2. Biological Material

The octocoral *I. hippuris* was collected by hand using scuba at Orchid Island, 70 km off the southeastern coast of Taiwan, in August 2008 at a depth of 9 m and stored in a freezer until extraction. The voucher specimen (LY-19) was identified by Prof. Chang-Feng Dai, National Taiwan University and deposited at the Department of Marine Biotechnology and Resources, National Sun Yat-sen University, Taiwan.

### 3.3. Extraction and Isolation

A specimen of octocoral *I. hippuris* (4.0 kg, wet weight) was minced and exhaustively extracted with acetone (3 × 3 L) at room temperature. The combined acetone extracts was then partitioned between H_2_O and EtOAc. The resulting EtOAc extract (25.6 g) was subjected to gravity silica gel 60 column chromatography (Si 60 CC) using *n*-hexane–EtOAc and EtOAc–MeOH of increasing polarity, to give 44 fractions. Fraction 28 (0.86 g), eluted with *n*-hexane–EtOAc (1:6), was further subjected to Si 60 CC (*n*-hexane–EtOAc, 5:3) to give 4 subfractions. A subfraction 28-2 (105 mg) was separated by a RP-18 flash column (MeOH–H2O, 75:25 to 100:0) to give four fractions. In turn, a subfraction 28-2-2, eluted with MeOH–H_2_O (80:20), was further purified by RP-18 HPLC (MeOH–H_2_O–MeCN, 80:20:5) to affford **1** (3.0 mg) and **4** (0.5 mg). Similarly, the subfraction 28-3 (112 mg) was further subjected to a RP-18 flash column (MeOH–H2O, 75:25 to 100:0) to give five subfractions. A subfraction 28-3-2 (112 mg), eluted with MeOH–H_2_O (70:30), was further purified by RP-18 HPLC (MeOH–H_2_O–MeCN, 75:25:5) to obtain **1** (0.2 mg) and **4** (0.3 mg). Likewise, the subfraction 28-3-3, eluted with MeOH–H_2_O (80:20), was purified by RP-18 HPLC (MeOH–H_2_O–MeCN, 75:25:5) to give **5** (1.2 mg). Fraction 29 (0.41 g), eluted with *n*-hexane–EtOAc (1:7), was subjected to Si 60 CC (*n*-hexane–EtOAc, 8:2 to 2:8) to give four subfractions. A subfraction 29-3 (309 mg), eluted with *n*-hexane–EtOAc (2:7), was further fractionated by a RP-18 flash column (MeOH–H2O, 60:40 to 100:0) to give four subfractions. A subfraction 29-3-2, eluted with MeOH–H_2_O (75:25), was further purified by RP-18 HPLC (MeOH–H_2_O, 70:30) to afford **3** (1.0 mg), **2** (1.2 mg), and **1** (0.2 mg).

**Hipposterone M** (**1**): White amorphous powder; [α]_D_ ^25^ −8 (*c* 0.1, CHCl_3_); IR (neat) ν_max_ 3454, 2954, 2922, 1733, 1717, 1558, 1456, 1374, 1238, 1152, 1019 cm^−1; 1^H NMR (CDCl_3_, 400 MHz) and ^13^C NMR (CDCl_3_, 100 MHz) data in Tables 1 and 2; HRESIMS *m/z* 599.3556 [M + Na]^+^ (calcd for C_33_H_52_O_8_Na, 599.3560).

**Hipposterone N** (**2**): White amorphous powder; [α]_D_ ^25^ −11 (*c* 0.1, CHCl_3_); IR (neat) ν_max_ 3463, 2970, 2933, 1731, 1715, 1374, 1244, 1021, 735 cm^−1; 1^H NMR (CDCl_3_, 400 MHz) and ^13^C NMR (CDCl_3_, 100 MHz) data in Tables 1 and 2; HRESIMS *m/z* 557.3452 [M + Na]^+^ (calcd for C_31_H_50_O_7_Na, 557.3454).

**Hipposterone O** (**3**): White amorphous powder; [α]_D_ ^25^ −5 (*c* 0.1, CHCl_3_); IR (neat) ν_max_ 3471, 2974, 2939, 1731, 1449, 1373, 1247, 1023, 739 cm^−1; 1^H NMR (CDCl_3_, 400 MHz) and ^13^C NMR (CDCl_3_, 100 MHz) data in Tables 1 and 2; HRESIMS *m/z* 657.3616 [M + Na]^+^ (calcd for C_35_H_54_O_10_Na, 657.3614).

**Hipposterol G** (**4**): White amorphous powder; [α]_D_ ^25^ +5 (*c* 0.1, CHCl_3_); IR (neat) ν_max_ 3471, 2928, 2860, 1734, 1454, 1371, 1244, 1023, 736 cm^−1; 1^H NMR (CDCl_3_, 500 MHz) and ^13^C NMR (CDCl_3_, 125 MHz) data in Tables 1 and 2; HRESIMS *m/z* 643.3819 [M + Na]^+^ (calcd for C_35_H_56_O_9_Na, 643.3822).

**Hppuristeroketal A** (**5**): White amorphous powder; [α]_D_ ^25^ +21 (*c* 0.1, CHCl_3_); IR (neat) ν_max_ 3471, 2974, 1731, 1373, 1248, 1041, 739 cm^−1; 1^H NMR (C_6_D_6_, 500 MHz) and ^13^C NMR (CDCl_3_, 125 MHz) data in Tables 1 and 2; HRESIMS *m/z* 645.3975 [M + Na]^+^ (calcd for C_35_H_58_O_9_Na, 645.3978).

### 3.4. Cytotoxicity Assay

Cytotoxicity was determined on P-388 (mouse lymphocytic leukemia), HT-29 (human colon adenocarcinoma), and A-549 (human lung epithelial carcinoma) tumor cells using a modification of the MTT colorimetric method according to a previously described procedure [19,20]. The provision of the P-388 cell line was supported by J.M. Pezzuto, formerly of the Department of Medicinal Chemistry and Pharmacognosy, University of Illinois at Chicago. HT-29 and A-549 cell lines were purchased from the American Type Culture Collection.

### 3.5. Anti-HCMV Assay

To determine the effects of natural products upon HCMV cytopathic effect (CPE), confluent human embryonic lung (HEL) cells grown in 24-well plates were incubated for 1 h in the presence or absence of various concentrations of tested natural products. Then, cells were infected with HCMV at an input of 1000 pfu (plaque forming units) per well of 24-well dish. Antiviral activity was expressed as IC_50_ (50% inhibitory concentration), or compound concentration required to reduce virus induced CPE by 50% after 7 days as compared with the untreated control. To monitor the cell growth upon treating with natural products, an MTT-colorimetric assay was employed [21].

## Figures and Tables

**Table 1:** ^13^C NMR data for compounds **1**–**5**.

C#	1, a*δ*_C_, type	2, a*δ*_C_, type	3, a*δ*_C_, type	4, b*δ*_C_, type	5, c*δ*_C_, type
1	38.3, CH_2_	38.3, CH_2_	38.2, CH_2_	36.7, CH_2_	35.6, CH_2_
2	38.1, CH_2_	38.1, CH_2_	38.0, CH_2_	31.4, CH_2_	29.2, CH_2_
3	211.7, qC	211.7, qC	211.5, qC	71.2, CH	100.7, qC
4	44.5, CH_2_	44.5, CH_2_	44.5, CH_2_	38.0, CH_2_	36.2, CH_2_
5	46.5, CH	46.5, CH	46.4, CH	44.7, CH	43.0, CH
6	28.6, CH_2_	28.5, CH_2_	28.5, CH_2_	28.3, CH_2_	28.8, CH_2_
7	31.7, CH_2_	31.7, CH_2_	31.5, CH_2_	31.9, CH_2_	32.7, CH_2_
8	34.5, CH	34.4, CH	34.4, CH	34.5, CH	35.1, CH
9	53.1, CH	53.6, CH	53.4, CH	54.0, CH	55.1, CH
10	35.7, qC	35.7, qC	35.6, CH	35.5, qC	36.4, qC
11	21.0, CH_2_	21.0, CH_2_	21.5, CH_2_	21.4, CH_2_	22.0, CH_2_
12	30.6, CH_2_	31.0, CH_2_	32.4, CH_2_	32.2, CH_2_	33.3, CH_2_
13	46.8, qC	46.7, qC	45.6, qC	45.6, qC	46.5, qC
14	47.7, CH	48.7, CH	49.2, CH	48.7, CH	50.3, CH
15	33.3, CH_2_	33.3, CH_2_	33.5, CH_2_	33.4, CH_2_	34.4, CH_2_
16	70.0, CH	70.1, CH	70.3, CH	70.1, CH	71.1, CH
17	80.0, qC	79.7, qC	77.2, qC	77.7,qC	78.6, qC
18	61.9, CH_2_	61.9, CH_2_	63.5, CH_2_	63.5, CH_2_	64.3, CH_2_
19	11.3, CH_3_	11.4, CH_3_	11.4, CH_3_	12.2, CH_3_	12.0, CH_3_
20	67.1, qC	67.5, qC	66.7, qC	66.4, qC	67.3, qC
21	16.1, CH_3_	15.9, CH_3_	16.1, CH_3_	16.2, CH_3_	17.1, CH_3_
22	77.2, CH	77.2, CH	77.2, CH	77.2, CH	78.1, CH
23	33.5, CH	32.9, CH	32.5, CH	33.6, CH	33.6, CH
24	39.9, CH	41.7, CH	38.8, CH	40.1, CH	42.2, CH
25	85.5, qC	73.7, qC	74.2, qC	85.6, qC	73.5, qC
26	23.2, CH_3_	30.9, CH_3_	71.0, CH_2_	22.8, CH_3_	31.2, CH_3_
27	25.1, CH_3_	25.8, CH_3_	20.3, CH_3_	25.1, CH_3_	25.9, CH_3_
28	10.4, CH_3_	11.4, CH_3_	10.9, CH_3_	10.5, CH_3_	11.7, CH_3_
29	11.9, CH_3_	12.1, CH_3_	12.3, CH_3_	11.9, CH_3_	12.6, CH_3_
OAc	20.9, CH_3_	20.9, CH_3_	21.2, CH_3_	21.2, CH_3_	21.1, CH_3_
	171.6, qC	171.6, qC	171.1, qC	171.0, qC	170.6, qC
	22.6, CH_3_		21.1, CH_3_	21.0, CH_3_	20.9, CH_3_
	169.8, qC		171.3, qC	171.2, qC	171.4, qC
			21.1, CH_3_	22.7, CH_3_	
			170.8, qC	169.9, qC	
OMe					47.6, CH_3_
					47.5, CH_3_

a Spectra were measured in CDCl_3_ (100 MHz);

b Spectra were measured in CDCl_3_ (125 MHz);

c Spectra were measured in C_6_D_6_ (125 MHz).

**Table 2:** ^1^H NMR data for compounds **1**–**5**.

H#	1, *δ*_H_ (*J* in Hz) a	2, *δ*_H_ (*J* in Hz) a	3, *δ*_H_ (*J* in Hz) a	4, *δ*_H_ (*J* in Hz) b	5, *δ*_H_ (*J* in Hz) c
1	α: 1.39 m	α: 1.35 m	α: 1.32 m	α: 1.02 m	α: 1.33 m
	β: 2.02 m	β: 2.00 m	β: 1.97 m	β: 1.69 m	β: 1.06 m
2	α: 2.32 m	α: 2.31 m	α: 2.31 m	α: 1.82 m	α: 1.86 m
	β: 2.38 m	β: 2.39 m	β: 2.37 m	β: 1.41 m	β: 1.41 m
3				3.60 m	
4	α: 2.12 dd ovl	α: 2.09 dd ovl	α: 2.12 dd ovl	α: 1.58 m	α: 1.86 dd (13.6, 3.6)
	β: 2.28 t (13.6)	β: 2.27 t (13.6)	β: 2.26 t (13.6)	β: 1.29 m	β: 1.41 dd ovl
5	1.56 m	1.54 m	1.55 m	1.54 m	1.34 m
6	1.38 m	1.38 m	1.39 m	1.34 m	1.08 m
7	1.79 m	1.78 m	1.82 m	1.78 m	1.54 m
	0.93 m	0.92 m	0.95 m	0.91 m	0.67 m
8	1.58 m	1.58 m	1.72 m	1.67 m	1.45 m
9	0.85 m	0.74 m	0.81 m	0.75 m	0.70 m
11	α: 1.66 m	α: 1.65 m	α: 1.63 m	α: 1.60 m	α: 1.48 m
	β: 1.48 m	β: 1.44 m	β: 1.33 m	β: 1.23 m	β: 1.23 m
12	α: 1.28 m	α 1.28 m	α: 1.34 m	α: 1.38 m	α: 1.44 m
	β: 2.44 m	β: 2.44 m	β: 2.16 m	β: 2.17 m	β: 2.28 m
14	1.36 m	1.18 m	1.23 m	1.31 m	1.28 m
15	α: 2.23 m	α: 2.24 m	α: 2.21 m	α: 2.21 m	α: 2.22 m
	β: 1.44 m	β: 1.46 m	β: 1.48 m	β: 1.46 m	β: 1.59 m
16	4.10 t (7.2)	4.13 t (7.6)	4.06 t (7.6)	4.04 dd (8.0, 7.5)	4.38 t (7.5)
18	3.75 t (10.4)	3.74 t (11.2)	4.23 d (11.6)	4.27 d (11.5)	4.55 d (11.5)
	3.94 d (11.6)	3.94 dd (11.6, 2.4)	4.30 d (11.6)	4.20 d (11.5)	4.49 d (11.5)
19	1.02 s	1.02 s	1.02 s	0.82 s	0.64 s
21	1.64 s	1.66 s	1.60 s	1.59 s	1.84 s
22	4.62 d (10.8)	4.60 d (10.8)	4.66 d (10.8)	4.67 d (11.0)	5.04 d (10.5)
23	2.28 m	2.47 m	2.50 m	2.29 m	2.43 m
24	1.97 q (8.0)	1.47 q (6.8)	1.64 q (6.8)	1.92 q (7.0)	1.55 q (7.5)
26	1.56 s	1.24 s	3.89 d (11.6)	1.56 s	0.88 s
			4.04 d (11.6)		
27	1.43 s	1.21 s	1.18 s	1.46 s	0.78 s
28	0.90 d (8.0)	0.90 d (6.8)	0.88 d (6.8)	0.91 d (7.0)	0.65 d (7.5)
29	0.88 d (6.4)	0.86 d (6.4)	0.88 d (6.8)	0.87 d (7.0)	0.80 d (7.0)
OAc	2.14 s, 1.99 s	2.14 s	2.07 s, 2.13 s, 2.13 s	2.06 s, 2.00 s, 2.13 s	1.76 s, 1.69 s
OMe					3.12 s, 3.02s
OH-16	3.36 s	3.43 s	3.27 br s	3.19 br s	3.83 br s
OH-18	2.44 d ovl	3.46 d ovl			

a Spectra were measured in CDCl_3_ (400 MHz);

b Spectra were measured in CDCl_3_ (500 MHz);

c Spectra were measured in C_6_D_6_ (500 MHz).
